# Defective transcription of ATF3 responsive genes, a marker for Cockayne Syndrome

**DOI:** 10.1038/s41598-020-57999-4

**Published:** 2020-01-24

**Authors:** Alexey Epanchintsev, Marc-Alexander Rauschendorf, Federico Costanzo, Nadege Calmels, Cathy Obringer, Alain Sarasin, Frederic Coin, Vincent Laugel, Jean-Marc Egly

**Affiliations:** 1 IGBMC, Department of Functional Genomics and Cancer, Equipe Labellisée Ligue 2014, CNRS/INSERM/University of Strasbourg, BP 163, 67404 Illkirch Cedex, C.U., Strasbourg, France; 2 Centre National de la Recherche Scientifique, UMR7104, 67404 Illkirch, France; 3 Institut National de la Santé et de la Recherche Médicale, U964, 67404 Illkirch, France; 4 Université de Strasbourg, 67404 Illkirch, France; 5 Laboratory of Medical Genetics, University of Strasbourg, 11 rue Humann, 67000 Strasbourg, France; 6 Department of Pediatric Neurology, Strasbourg University Hospital, Avenue Moliere, 67098 Strasbourg, Cedex France; 7 Laboratory of Genetic Instability and Oncogenesis UMR8200CNRS, Institut Gustave Roussy and University Paris-Sud, Villejuif, France; 8 Present Address: Molecular Health GmbH, Kurfürsten-Anlage 21, 69115, Heidelberg, Germany; 9 Present Address: Sanofi-Aventis Deutschland GmbH, Industriepark Höchst, 65926 Frankfurt, Germany

**Keywords:** Gene expression, Base excision repair, Transcription

## Abstract

Cockayne syndrome (CS) is a rare genetic disorder caused by mutations (dysfunction) in *CSA* and *CSB*. CS patients exhibit mild photosensitivity and severe neurological problems. Currently, CS diagnosis is based on the inefficiency of CS cells to recover RNA synthesis upon genotoxic (UV) stress. Indeed, upon genotoxic stress, *ATF3*, an immediate early gene is activated to repress up to 5000 genes encompassing its responsive element for a short period of time. On the contrary in CS cells, *CSA* and *CSB* dysfunction impairs the degradation of the chromatin-bound ATF3, leading to a permanent transcriptional arrest as observed by immunofluorescence and ChIP followed by RT-PCR. We analysed ChIP-seq of Pol II and ATF3 promoter occupation analysis and RNA sequencing-based gene expression profiling in CS cells, as well as performed immunofluorescence study of ATF3 protein stability and quantitative RT-PCR screening in 64 patient cell lines. We show that the analysis of few amount (as for example *CDK5RAP2, NIPBL* and *NRG1*) of ATF3 dependent genes, could serve as prominent molecular markers to discriminate between CS and non-CS patient’s cells. Such assay can significantly simplify the timing and the complexity of the CS diagnostic procedure in comparison to the currently available methods.

## Introduction

The human genome is constantly exposed to genotoxic attack, which might lead, in absence of proper DNA repair, to genomic instability thus resulting in rare disorders such as xeroderma pigmentosum (XP), trichothiodystrophy (TTD) and Cockayne syndrome (CS). CS patients exhibit photosensitivity, developmental and neurological abnormalities as well as progeroid features^1,2^ and could be ranged from the most severe prenatal and neonatal subtypes (COFS syndrome and CS type II) to infantile (CS type I) and juvenile subtypes (CS type III) leading to severe handicap and premature death. The CS incidence has been estimated in Western Europe at 2.7 cases per million births^3^. However, it seems that CS seems to be underdiagnosed. CS can be caused by mutations in *ERCC6* (*CSB*, [MIM: 609413]) or *ERCC8* (*CSA*, [MIM: 609412]) while certain mutations in *XPB* (MIM: 610651), *XPD* (MIM: 278730) and *XPG* (MIM: 278780) genes also develop some CS clinical features^4–7^. CS patient cells have a defect in transcription-coupled repair (TCR), a sub-pathway of Nucleotide Excision Repair (NER)^8^. CSB as well as XPB and XPD (two subunits of transcription factor TFIIH) and XPG appear likewise to play a role in general transcription^9–12^, suggesting that transcriptional defects are the underlying cause of growth and developmental abnormalities in CS. It has been hypothesized that RNA polymerase II (Pol II) stalled in front of a DNA lesion might be targeted by CSB and CSA to further recruit the other NER factors and proceed to eliminate the lesion from the transcribed strand^13,14^. However accumulating evidence pointed out their involvement in transcriptional regulation besides their defined role as TCR factors. Although defective DNA repair provided some explanation for the sun sensitivity phenotype, other clinical features of CS such as neurological dysfunction require additional explanations.

**Figure 1:**
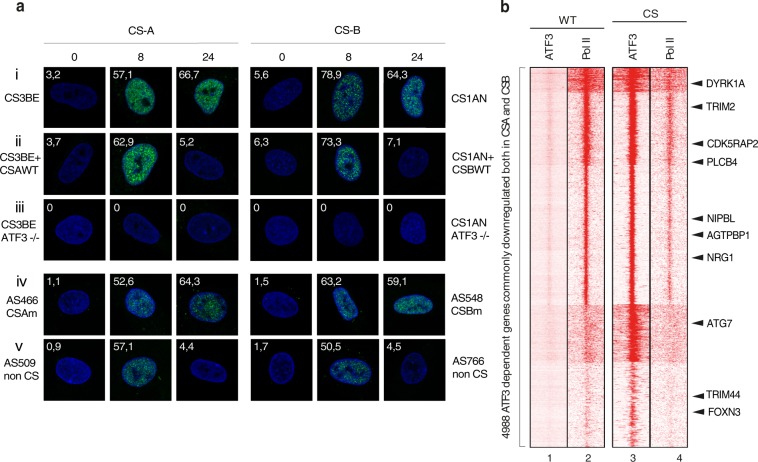
ATF3-dependent gene suppression upon genotoxic stress in patients CSA, CSB and non-CS cells. (**a**) Immunofluorescence showing ATF3 staining in CS3BE, CS3BE + CSAWT, CS3BE ATF3−/− cells (upper panel) and AS466, AS509 cells (lower panel) as well as the ATF3 staining in CS1AN, CS1AN + CSBWT, CS1AN ATF3−/− cells (upper panel) and AS548, AS766 cells (lower panel) The actual percentage of ATF3 positive cells is depicted for each condition. Several fields with on average 200 cells per condition were taken. UV-C irradiation 12 J/m^2^. (**b**) Heatmaps of ATF3 and Pol II ChIP sequencing 24 h after UV irradiation of CS1AN + CSBWT and CS1AN cells.

Despites the hypothesis suggesting the lesion-stalled Pol II as the main cause of transcription arrest, transcription of undamaged genes was also found to be dysregulated after genotoxic stress^15^. Indeed, we later have shown that genotoxic stress triggered the overexpression of immediate early genes such as the Activating Transcription Factor 3 (*ATF3*, MIM: 603148)^16^. ATF3 bound and repressed genes containing a CRE/ATF responsive element (Fig. 1 and Supplementary Fig. S1a) for a short period of time. The arrival of CSA and CSB as part of an ubiquitin machinery together with CUL4A and MDM2 ubiquitin ligases then triggered ATF3 elimination from its binding site^16,17^. On the contrary in CS cells, CSA and CSB dysfunction impaired ATF3 degradation. In these cells, ATF3 remained bound to the CRE/ATF3 response element, thereby preventing the comeback of Pol II and the restart of transcription (Fig. 1 and Supplementary Fig. S1a).

*ATF3*, an immediate early gene, overexpressed following cellular stress, is closely linked to motor and sensory neuron degeneration and sometimes used as a neuronal damage marker^18,19^. We thus hypothesized that ATF3 direct downstream transcriptional targets may be responsible for mediating CS clinical outcomes.

In this study, we show how the analysis of a certain set of ATF3 responsive genes help to discriminate between CS and non-CS cells. Indeed, in UV treated non-CS cells, ATF3 is overexpressed and targets the ATF/CRE responsive elements of a large set of genes to repress them for a short period of time. On the contrary in CS deficient cells, ATF3 is maintained on its binding site and the expression of its responsive genes is abrogated. Such assay focuses on the expression profile of only three of these genes such as *CDK5RAP2, NIPBL* and *NRG1* that could serve as prominent molecular markers to identify patients with CS phenotype.

## Methods

### Cell lines and UV-C treatment

All primary or immortalized fibroblasts were grown, expanded or subjected to analysis in DMEM/HamF10 (1:1) medium containing 10% FCS and 40 μg/ml gentamycin. Conditionally expressing WT-CSA CS3BE^20,21^, WT-CSB CS1AN and ATF3 knockout cells were originally described in^17^. To induce CSA or CSB expression, cells were exposed to growth medium containing Doxycycline (final concentration 0,5 μg/mL). For UV irradiation with a UV-C (254 nm) lamp the medium was removed and the cells were rinsed with PBS. After UV-C irradiation (12 J/m^2^) fresh medium was added and the cells were returned to the incubator for the times indicated in the figures.

All experiments on patient’s fibroblasts were conducted in accordance with French regulations. Informed consent for genetic assays was obtained from all patients or their legal guardians and the use of this human material for research purposes received a specific approval of the Local Ethics Committee (Comite de Protection des Personnes EST-IV).

### Immuno-staining

Cells were plated on coverslips and as soon as they rich proper density treated with UV and finally fixed 4% PFA. After permeabilization with triton-X cells were stained with anti-ATF3 antibody (Abcam, 44C3a). Images were acquired on confocal Leica SP8 microscope.

### ChIP assay

For ChIP assays, cells were seeded in 15 cm dishes and grown to subconfluency before irradiation with UV-C. A descriptive protocol for ChIP could be found elsewhere^22–24^. Antibodies were purchased or obtained from Santa Cruz: ATF3 (sc-188x), RNA Pol II (sc-9001x). The IP-ed DNA was subjected to purification and concentration by QIAquick PCR purification kit. For each time point, both, eluted IP or the initial input DNA were amplified by qPCR using “QuantiTect SYBR Green PCR MasterMix” kit using primer pairs flanking CRE/ATF or promoter/TSS region of respective gene. Primer oligonucleotides have been described in^16,17^.

### ChIP-seq, RNA-seq and NGS NGS sequencing of XP genes

Sample preparation for ChIP-seq and RNA-seq as well as NGS sequencing was described in^17,25^. The raw and processed ChIP-seq data are available at GEO: http://www.ncbi.nlm.nih.gov/geo/query/acc.cgi?acc=GSE87562. Gene expression was uploaded at GEO: http://www.ncbi.nlm.nih.gov/geo/query/acc.cgi?acc=GSE87540). Sample preparation and NGS sequencing of XP genes were described in the same way as in^26^. All primer sequences are available upon request.

### RNA isolation qRT-PCR analyses

Step-by-step protocol describing standard procedures of gene expression profiling attached as Supplementary Protocol.

## Results

### ATF3 regulation in CS deficient cells upon UV stress

To study the transcriptional response of CS cells following genotoxic attack, immuno-staining in CS3BE (CSA deficient) and CS1AN (CSB deficient) cells as well as in the corresponding rescued cells 24 h after UV irradiation (12 J/m^2^) was performed. In both CS3BE and CS1AN cells, ATF3 was still present 24 h post UV treatment, while in the CS1AN + CSB and CS3BE + CSA rescued cells, ATF3 appeared shortly peaking at 8 h and was gone by 24 h (Fig. 1a, upper panels). Similarly, the ATF3 cellular pattern at 24 h post UV irradiation, could discriminate between the CS patient’s AS466 (CSAm) and AS548 (CSBm) and non-CS patient’s fibroblasts (lower panels). As a control, ATF3−/− knockout cells showed no detection of ATF3 staining (middle panels).

RNA-Seq and ChIP-seq showed that both UV treated CSA and CSB deficient cells shared up to 70% (6,000) of commonly deregulated genes, most of them (85%) being targeted by ATF3^17^. UV-induced ATF3 was found occupying promoters of around 85% (4988 genes) of the down-regulated genes 24 h post UV treatment in both wild type and CS cells. Furthermore, heat maps of signal distribution from ChIP-seq, discriminate between ATF3 and Pol II following UV irradiation at genes encompassing a CRE/ATF element (Fig. 1b). In CS cells, ATF3 was still bound to chromatin 24 h post UV irradiation, preventing the recruitment of Pol II. Inversely, in WT cells, the disappearance of ATF3 at 24 h, allowed the proper arrival of Pol II at gene promoters to restart transcription.

Altogether, our data suggest that following genotoxic stress, the non-degradation of ATF3 by CSA and CSB, could serve a discriminative marker to distinguish CS among the other diseases.

### *CDK5RAP2, NIPBL* and *NRG1* as potential markers for CS patients

**Figure 2:**
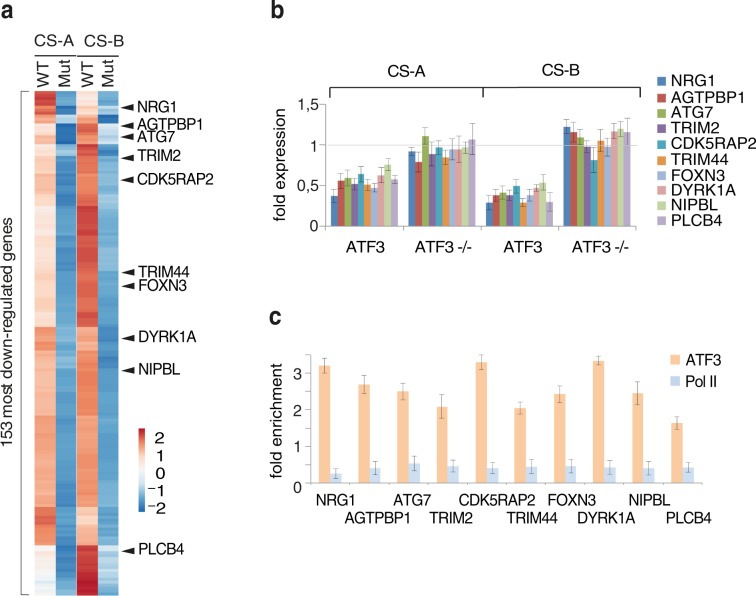
Expression profile in CS cells upon genotoxic stress. (**a**) RNA-seq gene expression profile of CS3BE + CSAWT, CS3BE, CS1AN + CSBWT and CS1AN cell lines. Data of the 153 most down-regulated genes in both CS3BE and CS1AN cells 24 hours after UV irradiation. At the right side of the panel are mentioned the 10 selected genes. (**b**) Quantitative RT-PCR analysis of the 10 selected ATF3-dependent genes in CSA (CS3BE) and CSB (CS1AN) deficient cells as well as respective ATF3−/− cells 24 hours after UV irradiation. Results are presented as the ratio of the value obtained at 24 relative to that of the untreated time (t = 0). Each point represents the average of at least 3 independent experiments. (**c**) ChIP assay determining the presence of ATF3 and Pol II at CRE/ATF sites of the 10 selected genes in CS1AN cells 24 hours after UV irradiation.

We have further analyzed original RNA-seq and ChIP-seq data and selected 153 (among the 4988) of the most down-regulated ATF3-dependent genes in both CSA and CSB cells (Fig. 2a, Supplementary Table S1); these genes showed complete recovery in CSA and CSB rescued (WT) cells within 24 hours. Further analyzing this pattern, we shortened the list and focused on 10 target genes, all of them encompassing the ATF3 binding site. Some of these genes, when mutated resulted in clinical features (including severe neurodegenerative ones) overlapping with those of CS (Table 1). RT-PCR experiments further showed that these genes failed to recover normal transcription activity 24 h post UV irradiation in both CS3BE and CS1AN cells as indicated by the ratio *of the value obtained at 24 relative to that of the untreated time (t* = *0)* (Fig. 2b). On the contrary, in UV treated CS3BE/ATF3−/− and CS1AN/ATF3−/− cells, these genes were not down regulated (compare ATF3 with ATF3−/−). Strikingly, ChIP experiments using antibodies against ATF3 and Pol II, further showed that promoters of these genes were still occupied by ATF3 in CS-A and CS-B cells 24 h after UV irradiation (Fig. 2c), thus abrogating the comeback of Pol II and the restart of transcription (see also^16,17^). We also tested 15 additional genes and found all of them being down-regulated in CS1AN (Supplementary Fig. S1b), meaning they were repressed by ATF3; the latter one being maintained on its CRE/ATF binding site.

**Table 1:** ATF3 target genes associated with genetic disorders.

Gene	MIM	Related disease
*NRG1*	#142445	Control meylination, neurodevelopment
*AGTPBP1*	#606830	Purkinje cell degeneration phenotype
*ATG7*	#608760	Purkinje axonal degeneration
*TRIM2*	#614141	Axonal nuropathies, neorodegeneration
*CDK5RAP2*	#608201	Microcephaly 3, primary, autosomal recessive
*TRIM44*	#612298	Predominant expression in cerebellum, cerebrum
*FOXN3*	#602628	
*DYRK1A*	#600855	Mental retardation, autosomal dominant 7
*NIPBL*	#608667	Cornelia de Lange syndrome 1
*PLCB4*	#600810	Retinal degeneration

For each gene is given the relative dysfunctional phenotype documented in MIM.

These findings prompted us to set up an assay to identify CS patients. We thus blindly tested 64 cell lines after being UV treated by considering three genes with a reliable biological relevance to CS (Table 1): (i) *CDK5RAP2* is involved in brain development^27,28^; dysfunctional alleles of *CDK5RAP2* are associated with autosomal recessive primary microcephaly (*MCPH3*^27^; (ii) *NIPBL* mutations result in Cornelia De Lange syndrome (MIM: 122470), which is characterized by smaller brain size resulted from impaired neuron production in the developing cortex^29^; (iii) *NRG1* encodes for a multifunctional neurotrophin that mediates neurodevelopment. *NRG1* signaling in oligodendrocytes has indeed been involved in prefrontal cortex myelination^30,31^ and peripheral myelination^32^ resulting in defect in normal cognitive function^33^. Some of these features overlap the clinical picture of CS. RT-PCR next showed that *CDK5RAP2*, *NRG1* and *NIPBL* as well as *DHFR*, here used as a standard^16^ all of them encompassing a CRE/ATF binding site and being ATF3 responsive genes, were downregulated and unable to recover normal RNA synthesis past 24 hours post UV irradiation in both CS3BE and CS1AN cells (Supplementary Fig. S1c, the kinetic of expression of these genes). On the contrary, in CS3BE + CSA-WT and CS1AN CSB-WT rescued cells, these genes that were down regulated few hours after UV treatment, recovered their normal expression past 24 hours.

Hence, genotoxic attack in CS cells induces a reproducible downregulation of ATF3 dependent genes that, when defective, were shown to be responsible of features resembling CS phenotype.

### Identification of the CS phenotype

**Figure 3:**
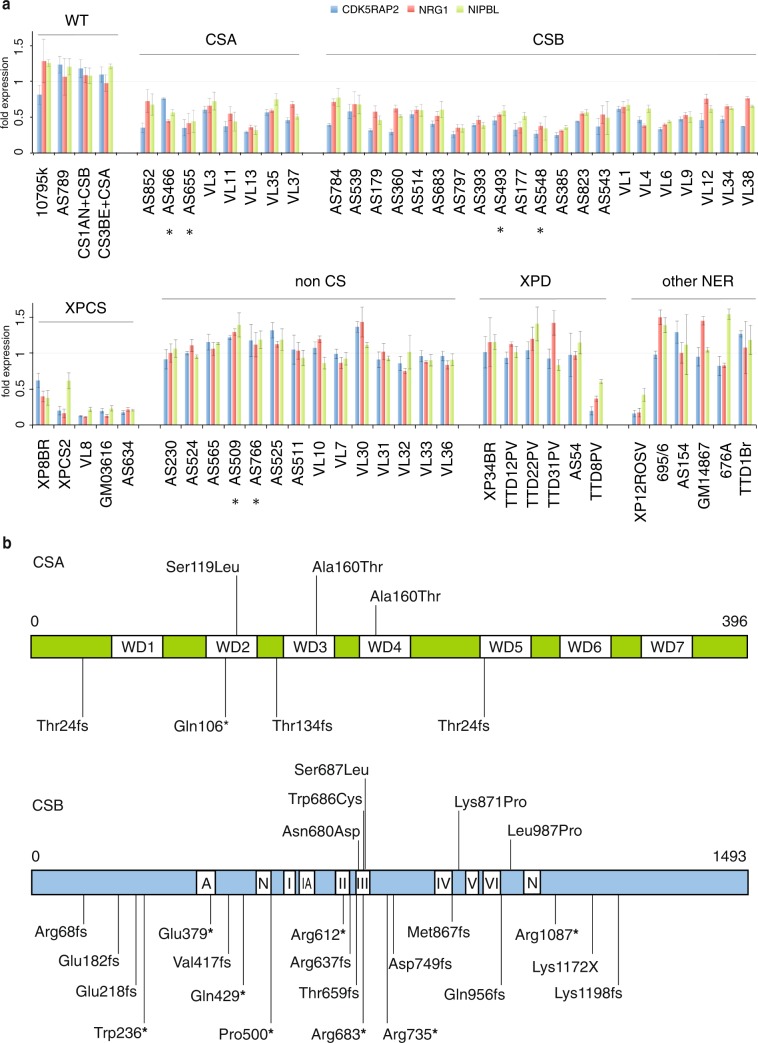
Blind assay: mRNA expression patterns in patient-derived primary fibroblasts. (**a**) Quantitative RT-PCR analysis of the *CDK5RAP2, NIPBL* and *NRG1* ATF3 dependent genes in CSA, CSB, non-CS patients. All the measurements represent a comparison between 0 and 24 hours time point after UV treatment. All the 64 cells were grown in parallel and UV (12 J/m^2^) irradiated; RT-PCR was done on cDNA from cells collected 0 and 24 hours after UV irradiation. Values are presented as fold expression in relation to the internal expression control *GAPDH* and to expression level of each gene at time point t = 0 (w/o UV irradiation). (**b**) Maps of CSA and CSB proteins showing all the mutations involved in this study (Supplementary Table S2).

We next wondered if a simple screening of *CDK5RAP2, NRG1* and *NIPBL* would be sufficient to identify CS phenotype among the 64 blindly tested cell lines (summarized in Fig. 3a and Supplementary Fig. S1d). Out of these cell lines, 29 were diagnosed as CS: 8 and 21 with mutations in *CSA* and *CSB* respectively, were associated with CS type I, II, III and COFS syndrome (Fig. 3b and Supplementary Table S2)^1^. We observed a clear decrease in the expression profile of the three genes for the 29 CS cell lines compared to the non-CS. No difference has been shown between CSA and CSB cell lines, neither between the cell lines derived from patients with various levels of clinical severity (CS type I, type II and type III).

Among the cells that recovered a normal RNA synthesis 24 h past UV irradiation, (i) 4 were wild type, (ii) 14 patients were named “non-CS patients”. These patients included in the blind assay, presented overlapping clinical features (microcephaly, growth retardation and mental disability) with CS but did not show any mutations in NER genes nor any DNA repair defect in functional assays. In some of these cases, a mitochondrial defect or others neurodegenerative disorders were suspected, (iii) 10 were cell lines from patients with known mutations in *XPB*, *XPD*, *TTD-A/p8*, *XPA* (MIM: 278700), *XPC* (MIM: 78720) or *XPF* (MIM: 278760); All cells derived from patients who only showed classical dermatological symptoms (either pure TTD phenotype or pure XP phenotype) as well as an *XPA* silenced cell line (695/6, shXPA^34^ displayed a normal transcription profile for the three tested genes (*CDK5RAP2, NRG1* and *NIPBL)*. To show that others among the downregulated genes were able to give the same expression signature, we tested other 6 genes instead of three to discriminate CS versus non-CS cells and got similar result (Supplementary Fig. S1g and S1h). We also observed a reduced expression profile for VL8 and AS634 cells (previously defined as non-CS) derived from patients characterized by microcephaly, growth failure, and cutaneous sensitivity and pigmentary anomalies. Sequencing showed mutations in ERCC5/XPG and ERCC2/XPD, prompting the diagnosis towards XP-G/CS for VL8 and as XP-D/CS for AS634 cells. In addition, cells from patients presenting the combined CS symptoms (XP/CS patients with mutation in *XPD* (XP8BR, XPSC2^35^) and *XPG* (GM03616^36^, VL8, AS634) showed a severe down-regulation of the three marker genes, similar to the VL8 and AS634 cell lines. The same pattern was also identified in UV treated cells from a patient previously characterized as severe-TTD (TTD8PV^37^ showing a diffuse demyelination of the cerebral white matter reminiscent of CS^38^) and a XP-A patient (XP12ROSV^39^) characterized by De Sanctis–Cacchione phenotype (dermatological symptoms of XP, associated with neurological deterioration and growth failure). In these two cell lines and similarly to what was observed in XP/CS cases, the down regulation of the three marker genes was again associated with clinical features reminiscent of the CS phenotype. As a further control, ChIP experiments showed that in all the CSA/CSB deficient cells as pointed out in AS493, AS548, AS179, AS655, XPCS2 (as * noted Supplementary Fig. S1e), ATF3 was recruited and remained bound to the CRE/ATF site throughout the entire 24 h time course following UV treatment. In some other cell lines (AS766, AS509, AS525) where gene expression remained unchanged after UV treatment, ATF3 remained recruited for a short period after which RNA synthesis was restored.

Although the same results were observed with larger panel of chosen candidates, screening of only three genes seems to be sufficient to identify and diagnose CS phenotype.

## Discussion

The present study proposes an easy and straightforward molecular assay for identifying CS patients based on the inability of CSA and CSB to perform their ubiquitin/proteasome degradation function. In response to cellular stress, ATF3 immediate early gene is activated and rapidly transiently targets genes harboring a CRE/ATF binding site to repress their expression. In CS deficient cells, the ATF3 is maintained on its target site, thus abrogating the expression of amount of ATF3 responsive genes leading to subsequent cellular defects as exemplified in our blind assay (Fig. 3a and Supplementary Fig. S1d). Immuno-staining using anti ATF3 antibodies might also quickly discriminate between CS and non-CS cells: in the first set of cells, ATF3 was visible at 24 hours while in the latter ones, ATF3 was eliminated few hours post UV irradiation (Fig. 1a).

Although that practically any of the top 153 ATF3 responsive genes could have served as markers, we focused on *CDK5RAP2, NRG1* and *NIPBL*, three genes involved in neurodevelopmental or neurodegenerative processes. The same pattern is indeed observed in all proven CS patients (all of them showing neurological symptoms (Fig. 3 and Supplementary Table S2)^1,2^ but also in patients carrying mutations in XP genes whenever these mutations are associated with neurological symptoms. However, this gene expression pattern is not observed in patients carrying mutations in XP genes, when these mutations are associated with dermatological symptoms only or in patients showing neurological defects but no mutation in the CS or XP genes. This transcriptional pattern could therefore be regarded as a molecular signature for CS. This could help to diagnose CS even before the identification of the molecular defect or in the absence of identified mutations in the known CS genes, beside already existing DNA repair tests. In addition, our assay could lead to the identification of patients presenting similar neurological phenotypes and transcriptional profile with no visible defects in DNA repair. Indeed, the clinical sensitivity to sunlight is usually mild in CS patients and is actually not a prominent feature of the clinical picture. When clinically assessed by dermatological photo-tests, a vast majority of CS patients are defined as photosensitive^40^ but the clinical relevance and awareness of this sun sensitivity in the everyday life of the patients largely depends on the country they live in and on their way of life (which can imply various levels of sun exposure). This is in sharp contrast with the very severe sensitivity to UV observed in CS cells. Neither the classical RRS assay nor the present assay is correlated with the clinical picture of CS patients: the most severe and the mildest CS patients have a similar RRS defect at the cellular level. Although there is a trend pointing to a more severe defect in XP-CS patients than in CS patients, there is no clear difference between CSA and CSB patients nor between CS type I (classical), type II (severe) or type III (mild)^1^. Our results also indicate that the present assay could also suggest the diagnostic of CS, regardless of the gene involved (*CSA, CSB, XPB, XPD, XPG*). This assay actually relies on the same paradigm as the classical RRS (it evaluates the restart of transcription after UV irradiation) but focuses only on the transcription of a more specific (ATF3 responsive) subset of clinically relevant genes rather than on the global transcription of the genome. In fact, our assay investigates the function of CSA and CSB in the regulation of gene expression upon genotoxic stress. In a clinical purpose however, it seemed relevant to validate markers that could be easily and unambiguously tested in fibroblasts, which are one of the only cell types that is available in clinical routine for obvious ethical issues.

We believe that our assay would also be clinically more relevant for diagnostic purposes than the classical DNA repair tests which were unable to discriminate neurological and non-neurological phenotypes linked to mutations in the NER genes. Of course it would be over simplistic to pretend that the few markers genes used here can alone account for pathophysiology of CS. Our assay might help to clarify overlapping NER clinical subtypes even in the absence of any identified molecular defect or in the very early stages of the disease although we are not excluding defects due to DNA lesions originated by UV irradiation which gets masked by the pool of ATF3 dependent transcription arrest.

## Supplementary information

Supplementary Figure and Tables.

## References

[1] Laugel V (2010). Mutation update for the CSB/ERCC6 and CSA/ERCC8 genes involved in Cockayne syndrome. Human mutation.

[2] Brooks PJ (2013). Blinded by the UV light: how the focus on transcription-coupled NER has distracted from understanding the mechanisms of Cockayne syndrome neurologic disease. DNA repair.

[3] Kleijer WJ (2008). Incidence of DNA repair deficiency disorders in western Europe: Xeroderma pigmentosum, Cockayne syndrome and trichothiodystrophy. DNA repair.

[4] Davies AA, Friedberg EC, Tomkinson AE, Wood RD, West SC (1995). Role of the Rad1 and Rad10 proteins in nucleotide excision repair and recombination. The Journal of biological chemistry.

[5] Sugasawa K (1998). Xeroderma pigmentosum group C protein complex is the initiator of global genome nucleotide excision repair. Molecular cell.

[6] Friedberg EC (1996). Cockayne syndrome–a primary defect in DNA repair, transcription, both or neither?. BioEssays: news and reviews in molecular, cellular and developmental biology.

[7] Bootsma GP, Dekhuijzen PN, van Herwaarden CL (1998). Effects of inhaled corticosteroids on bone. Neth J Med.

[8] Hanawalt PC (1994). Transcription-coupled repair and human disease. Science.

[9] Selby CP, Sancar A (1997). Human transcription-repair coupling factor CSB/ERCC6 is a DNA-stimulated ATPase but is not a helicase and does not disrupt the ternary transcription complex of stalled RNA polymerase II. The Journal of biological chemistry.

[10] Lee SK, Yu SL, Prakash L, Prakash S (2002). Yeast RAD26, a homolog of the human CSB gene, functions independently of nucleotide excision repair and base excision repair in promoting transcription through damaged bases. Molecular and cellular biology.

[11] Ito S (2007). XPG stabilizes TFIIH, allowing transactivation of nuclear receptors: implications for Cockayne syndrome in XP-G/CS patients. Molecular cell.

[12] Compe E, Egly JM (2016). Nucleotide Excision Repair and Transcriptional Regulation: TFIIH and Beyond. Annual review of biochemistry.

[13] Hanawalt PC, Spivak G (2008). Transcription-coupled DNA repair: two decades of progress and surprises. Nature reviews Molecular cell biology.

[14] Laine JP, Egly JM (2006). Initiation of DNA repair mediated by a stalled RNA polymerase IIO. The EMBO Journal.

[15] Proietti-De-Santis L, Drane P, Egly J-M (2006). Cockayne syndrome B protein regulates the transcriptional program after UV irradiation. Embo J.

[16] Kristensen U (2013). Regulatory interplay of Cockayne syndrome B ATPase and stress-response gene ATF3 following genotoxic stress. Proceedings of the National Academy of Sciences of the United States of America.

[17] Epanchintsev A (2017). Cockayne’s Syndrome A and B Proteins Regulate Transcription Arrest after Genotoxic Stress by Promoting ATF3 Degradation. Molecular cell.

[18] Gitiaux C (2015). Progressive demyelinating neuropathy correlates with clinical severity in Cockayne syndrome. Clinical neurophysiology: official journal of the International Federation of Clinical Neurophysiology.

[19] Sasaki K (1992). Demyelinating peripheral neuropathy in Cockayne syndrome: a histopathologic and morphometric study. *Brain &*. development.

[20] Ridley AJ, Colley J, Wynford-Thomas D, Jones CJ (2005). Characterisation of novel mutations in Cockayne syndrome type A and xeroderma pigmentosum group C subjects. Journal of human genetics.

[21] Nardo T (2009). A UV-sensitive syndrome patient with a specific CSA mutation reveals separable roles for CSA in response to UV and oxidative DNA damage. Proceedings of the National Academy of Sciences of the United States of America.

[22] Drane P, Compe E, Catez P, Chymkowitch P, Egly JM (2004). Selective regulation of vitamin D receptor-responsive genes by TFIIH. Molecular cell.

[23] Le May N (2010). NER factors are recruited to active promoters and facilitate chromatin modification for transcription in the absence of exogenous genotoxic attack. Molecular cell.

[24] Chymkowitch P, Le May N, Charneau P, Compe E, Egly JM (2011). The phosphorylation of the androgen receptor by TFIIH directs the ubiquitin/proteasome process. The EMBO Journal.

[25] Catic A (2013). Genome-wide map of nuclear protein degradation shows NCoR1 turnover as a key to mitochondrial gene regulation. Cell.

[26] Calmels N (2016). Uncommon nucleotide excision repair phenotypes revealed by targeted high-throughput sequencing. The Orphanet Journal of Rare Diseases.

[27] Fong KW (2009). Interaction of CDK5RAP2 with EB1 to track growing microtubule tips and to regulate microtubule dynamics. Mol Biol Cell.

[28] Barrera JA (2010). CDK5RAP2 regulates centriole engagement and cohesion in mice. Dev Cell.

[29] Tonkin ET, Wang TJ, Lisgo S, Bamshad MJ, Strachan T (2004). NIPBL, encoding a homolog of fungal Scc2-type sister chromatid cohesion proteins and fly Nipped-B, is mutated in Cornelia de Lange syndrome. Nature genetics.

[30] Michailov GV (2004). Axonal neuregulin-1 regulates myelin sheath thickness. Science.

[31] Taveggia C (2005). Neuregulin-1 type III determines the ensheathment fate of axons. Neuron.

[32] Garratt AN, Voiculescu O, Topilko P, Charnay P, Birchmeier C (2000). A dual role of erbB2 in myelination and in expansion of the schwann cell precursor pool. Journal of Cell Biology.

[33] Makinodan M, Rosen KM, Ito S, Corfas G (2012). A critical period for social experience-dependent oligodendrocyte maturation and myelination. Science.

[34] Alekseev S (2014). A small molecule screen identifies an inhibitor of DNA repair inducing the degradation of TFIIH and the chemosensitization of tumor cells to platinum. *Chemistry &*. biology.

[35] Theron T (2005). Transcription-associated breaks in xeroderma pigmentosum group D cells from patients with combined features of xeroderma pigmentosum and Cockayne syndrome. Molecular and cellular biology.

[36] Arlett CF, Priestley A (1983). Defective recovery from potentially lethal damage in some human fibroblast cell strains. International journal of radiation biology and related studies in physics, chemistry, and medicine.

[37] Botta E (1998). Analysis of mutations in the XPD gene in Italian patients with trichothiodystrophy: site of mutation correlates with repair deficiency, but gene dosage appears to determine clinical severity. The American Journal of Human Genetics.

[38] Battistella PA, Peserico A (1996). Central nervous system dysmyelination in PIBI(D)S syndrome: a further case. Child’s nervous system: ChNS: official journal of the International Society for Pediatric Neurosurgery.

[39] Kobayashi T (1998). Mutational analysis of a function of xeroderma pigmentosum group A (XPA) protein in strand-specific DNA repair. Nucleic acids research.

[40] Frouin E, Laugel V, Durand M, Dollfus H, Lipsker D (2013). Dermatologic findings in 16 patients with Cockayne syndrome and cerebro-oculo-facial-skeletal syndrome. JAMA Dermatol.

