# Supplementary material for: Defective transcription of ATF3 responsive genes, a marker for Cockayne Syndrome
Source: Sci Rep. 2020 Jan 24;10:1105. doi: 10.1038/s41598-020-57999-4 (PMC6981198; doi:10.1038/s41598-020-57999-4)
Supplement: Supplementary file 1 — Supplementary Figure and Tables. [file 41598_2020_57999_MOESM1_ESM.pdf]

Supplementary information

Defective transcription of ATF3 responsive genes, a marker for Cockayne Syndrome

Alexey Epanchintsev, Marc-Alexander Rauschendorf, Federico Costanzo, Nadege Calmels, Cathy Obringer, Alain Sarasin, Frederic Coin, Vincent Laugel and Jean-Marc Egly

Supplementary Figure S1

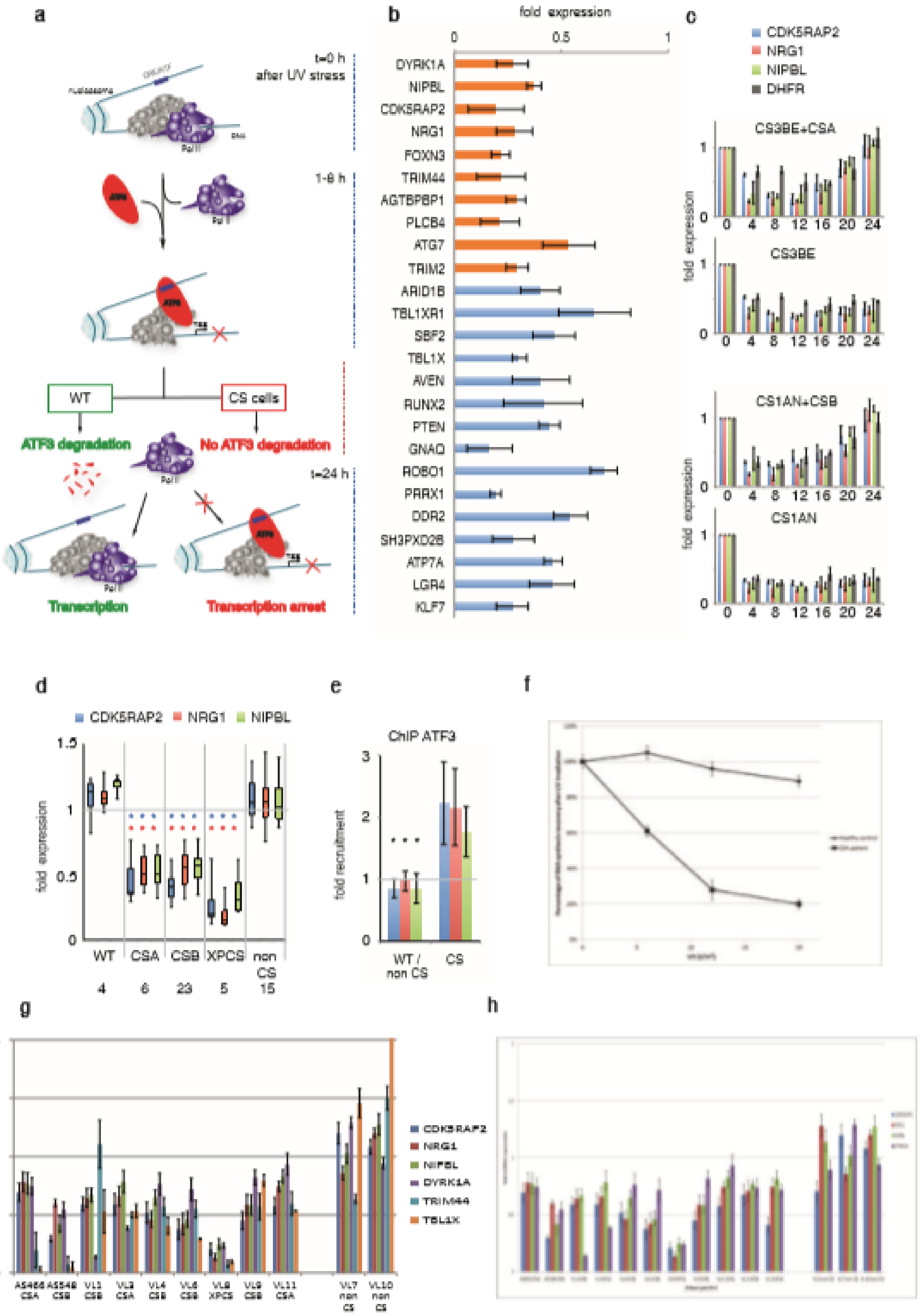

Supplementary Figure S1

(**a**) Model describing the fate of ATF3 in WT and CS deficient cells upon genotoxic attack. The neo-synthesized ATF3 targets the CRE/ATF site of its responsive genes while Pol II is released from the promoter. In WT cells, ATF3 was eliminated by a ubiquitination/proteasomal degradation process to allow the come-back of Pol II. In CS deficient cells, ATF3 is maintained at the promoter, since its degradation is prevented by mutations in either *CSA* or *CSB*[17].

(**b**) Quantitative RT-PCR analysis of the primary selection of the 25 ATF3-dependent genes in CSB (CS1AN) deficient cells 24 hours after UV irradiation selected from the list of the top 153 downregulated genes (**Supplementary Table S1**).

(**c**) An overtime expression of ATF3-dependent genes (*CDK5RAP2, NRG1, NIPBL* and *DHFR*) in CS3BE and CS1AN and corresponding rescued cells cell after UV irradiation.

(**d**) The summary of gene expression profile of (**Figure 3a**) depicted in box-and-whiskers plots for each mentioned population and each of three genes**.** Statistical differences between chosen populations for each gene is shown as a blue star in relation to WT and as a red star in relation to non-CS population (**Supplementary Table S3**).

(**e**) ChIP assay showing the constitutive ATF/CRE site occupation by ATF3 within 24 hours post UV treatment in CS background population, controversially to the WT/non-CS cell lines population described Figure 3a. Fold of ATF/CRE sites occupation measured by qPCR and represent a relation between 24h and 0h time points. Expression analysis was performed with at least three independent experiments and in triplicates.

(**f**) Dose response to UV-C irradiation applied on CSA and wtCSA restored cells. A relative density of survived cells was measured in 24 hours.

(**g**) Quantitative RT-PCR analysis of the *CDK5RAP2*, *NRG1, NIPBL, DYRK1A, TRIM44* and *TBLX1* ATF3 dependent genes in CSA, CSB, non-CS patients primary fibroblasts. Values are presented as fold expression in relation to the internal expression control *GAPDH* and to expression level of each gene at time point t=0 (w/o UV irradiation).

(**h**) Quantitative RT-PCR analysis of the *CDK5RAP2*, *NRG1*, *NIPBL* and *DYRK1A* ATF3 dependent genes in CSA, CSB, non-CS patients primary fibroblasts. Values are presented as fold expression in relation to the internal expression control *GAPDH* and to expression level of each gene at time point t=0 (w/o UV irradiation).

Supplementary Table S1. List of 153 the most down-regulated genes in CSA and CSB.

	CSA	CS3BE	CSB	CS1AN
ACSL3	0,86188225	0,47742455	0,95586874	0,45998967
ACTC1	0,52182854	0,31188786	0,84385355	0,32605211
ADA	1,03129806	0,46936833	0,90538622	0,48862222
AGTPBP1	0,71213465	0,4566301	0,83578621	0,63263536
AKAP13	0,80161678	0,4913785	0,86772755	0,46577686
ANKRD28	0,82946558	0,41003685	0,83058161	0,45011202
ARFGEF1	0,90475987	0,49133962	0,92798088	0,48305749
ARHGAP10	0,87221826	0,45092232	0,90152044	0,46290951
ARHGAP21	0,6406858	0,49248201	0,76618548	0,42119615
ARHGAP24	0,70278728	0,46454873	0,88293612	0,48292528
ARHGEF10L	0,93827753	0,45911317	0,9677391	0,48669915
ARID1B	0,66739908	0,41770417	0,77274209	0,42681665
ARSJ	0,60529545	0,39226382	0,81090293	0,48503111
ATG7	0,84200561	0,50494385	0,8753555	0,61826731
ATP7A	1,07918959	0,46182142	0,89645366	0,46682229
AVEN	0,81455762	0,46593957	0,85065317	0,46349205
BICC1	0,77422476	0,42770875	0,83945227	0,47580726
BMPR1A	0,9214489	0,46556933	0,92704222	0,48952238
CBLB	0,69573613	0,43553911	0,87181383	0,45767141
CDH11	0,70868765	0,36527245	0,77644436	0,45164834
CDK5RAP2	0,84345691	0,53185799	0,90435324	0,59800716
CUTC	1,22197536	0,45051	0,97976517	0,48673677
DAB2	0,56384002	0,43606122	0,86173175	0,4187741
DDR2	0,73267145	0,49374952	0,81458186	0,4824524
DOCK11	0,82392935	0,39992551	0,85653612	0,42009291
DYRK1A	0,95716511	0,57254964	0,95854895	0,48859736
EBF1	0,59849228	0,49752258	0,62510979	0,47663267
EFNA5	0,83164246	0,48724286	0,91748137	0,47299567
ENOX1	0,58271154	0,37617906	0,64936732	0,46901351
ERBB2IP	0,72045716	0,36101399	0,8531968	0,46188025
FAF1	0,72410339	0,41293442	0,82095629	0,47588292
FAM110B	0,78110245	0,43947005	0,74049058	0,3668912
FAM168A	0,71894138	0,42348296	0,85547921	0,47239274
FAM69A	0,74846439	0,4499293	0,79235305	0,35627989
FANCC	0,77205003	0,44753833	0,83175777	0,44611309
FBN1	0,58845614	0,47006427	0,71726385	0,45062322
FBXL7	0,49461435	0,32743872	0,63859959	0,41092595
FOXN3	0,72096605	0,36546878	0,85563266	0,38978051
FUT8	0,77912363	0,42081786	0,82093257	0,42371186
GALNT15	0,60167152	0,43961829	0,83527109	0,49066172
GNAQ	0,8358787	0,4875643	0,84230657	0,47871116

GNPTAB	0,84547942	0,48340855	0,90518758	0,48136621
GRB10	0,71413605	0,44573241	1,16928048	0,38485995
GRIN2A	0,62233752	0,47196553	0,61134036	0,44569788
GSK3B	0,78420865	0,47171712	0,89629709	0,46450684
HEG1	0,6209752	0,37997671	0,79570728	0,40017597
HIP1	0,7141697	0,42958014	0,90302464	0,4231034
IGF2BP2	0,72812627	0,41489667	0,87854844	0,49364669
JAZF1	0,61739166	0,36648112	0,82972089	0,48874773
KAZN	0,92376613	0,42603405	1,00319909	0,44704721
KLF12	0,63044556	0,36064573	0,65998758	0,39623974
KLF7	0,64063885	0,38740302	0,86036411	0,48935859
LDB2	0,47791111	0,27610506	0,5711697	0,38205208
LGR4	0,65273723	0,41288278	0,80307976	0,49655556
LMNB1	0,90644749	0,45521769	0,86144784	0,47468047
LPAR1	0,94346461	0,45379385	0,92061692	0,4797648
LPHN2	0,74525474	0,40570733	0,82046774	0,37705032
LRRC16A	0,7199878	0,38328284	0,87453924	0,40784926
LRRC20	0,7744103	0,43108986	0,76346354	0,43035329
LYPD6	0,87720373	0,37900793	1,26275656	0,43819071
MAP2K5	0,75790691	0,39311898	0,83892315	0,46142737
MAP4K3	0,99462964	0,46833015	0,94269108	0,44418382
MBNL2	0,78730176	0,44126153	0,81957517	0,48825188
MED13L	0,77273147	0,4645971	0,93943453	0,45022017
MEIS1	0,82961064	0,42190823	0,84091899	0,44352207
MEIS2	0,81839584	0,40114472	0,87548619	0,46115429
MELK	0,95139436	0,47675424	0,96848588	0,47251015
NDST1	0,72444498	0,45247496	0,82954795	0,44745791
NIPBL	0,86980146	0,53705394	0,92283554	0,51856392
NNMT	0,92538199	0,43753856	0,86301667	0,39008375
NOG	0,95883295	0,66596211	1,23426519	0,86689528
NREP	0,58553776	0,29074882	0,74452604	0,43603272
NRG1	0,94177899	0,45330528	0,86113844	0,62886811
NXN	0,89292348	0,40429674	0,80485094	0,45593845
PARD3	0,74188018	0,46053534	0,83584793	0,46812372
PBX3	0,7764625	0,45049615	0,8034517	0,49807556
PCCA	0,61865708	0,43156736	0,73108979	0,47637875
PDE6D	0,97362286	0,47127953	1,03101347	0,48805787
PDGFC	0,63135495	0,33150355	0,71832669	0,39609336
PDS5A	0,95439951	0,47572646	0,81574076	0,46267311
PDS5B	0,92440438	0,48738861	1,00046455	0,48963196
PEX14	0,92415161	0,46139007	0,91643026	0,49390967
PHF3	1,00423078	0,48396108	1,01152202	0,45972432
PHLPP1	0,78773977	0,48131795	0,93575993	0,43675449

PIK3CB	0,9261746	0,39481049	0,89777895	0,47813323
PKP4	0,79839413	0,46091201	0,890894	0,48777845
PLCB4	0,81448015	0,53620985	1,09173128	0,7220306
PPARA	0,70332049	0,46843772	0,84076853	0,487457
PPM1H	0,80871065	0,48275076	0,81548106	0,39460464
PPP2R5E	0,87806053	0,48172436	0,89691143	0,49584464
PRKD1	0,58994147	0,36713275	0,75737119	0,44096048
PRR16	0,64004689	0,35258559	1,0217976	0,49123714
PRRX1	0,82446216	0,40270469	0,77268653	0,43206935
PSD3	0,62108442	0,48928582	0,71338273	0,41118956
PTEN	0,88373098	0,44681692	0,8873655	0,43363889
PTP4A3	1,09981286	0,45005387	0,81142215	0,49059839
R3HDM2	0,88710778	0,44269247	0,82484414	0,45031661
RHOBTB1	0,69704424	0,46224072	0,77482223	0,45234877
RHOT1	0,77499738	0,44140839	0,85244656	0,43587598
RNF144B	0,73915706	0,47306728	0,9513038	0,48560747
RNF150	0,60805605	0,35175815	0,71598595	0,36788257
ROBO1	0,67278288	0,44492093	0,68272466	0,48084549
ROCK2	0,85519543	0,46972002	0,92105151	0,48699282
RPS6KC1	0,9523276	0,40800614	1,24323417	0,46533942
RSRC1	0,73735365	0,45246004	0,74418618	0,4681563
RUNX2	0,79267222	0,39773215	0,86934635	0,48686572
RXRA	0,87604845	0,48867209	0,96249691	0,48289602
SAMD12	0,62736058	0,39391961	0,72726216	0,41491499
SBF2	0,56859842	0,3849515	0,75335596	0,48789281
SEC24D	0,71640446	0,40836149	0,91280037	0,47475938
SH3PXD2B	0,71146408	0,48805511	0,8719548	0,4851238
SHROOM2	0,91680718	0,48943972	0,72230296	0,48777934
SIM1	0,71412406	0,38913615	0,9166233	0,46466729
SLC12A8	0,77181244	0,30789208	0,90372072	0,40156647
SLC7A1	0,70172566	0,49233566	0,88937976	0,49335279
SNTB1	0,61729802	0,44370794	0,63381293	0,35563309
SNX29	0,63821954	0,47154626	0,62332473	0,49485483
SOGA2	0,76616686	0,45859435	0,96560986	0,48164555
ST6GALNAC5	0,67646944	0,37429622	0,77242053	0,4703377
STXBP6	0,63867759	0,36771124	0,88031245	0,41651767
TANC2	0,63045729	0,39414906	0,7126653	0,40610286
TBL1X	0,73949117	0,41747923	0,77189504	0,42905366
TBL1XR1	0,87278963	0,46699965	0,77952598	0,48123889
TCF12	0,67068906	0,44380992	0,77091277	0,45814483
TCF4	0,73717868	0,40647119	0,83644442	0,44980752
TGFBR3	0,72243711	0,410351	0,7840661	0,40617545
THAP8	1,22862932	0,45914421	0,91049312	0,43065633

TMCC1	0,78493674	0,49212898	0,83519101	0,44860533
TMEFF2	0,69681604	0,42906639	0,6840164	0,38219832
TNS3	0,68495569	0,36255356	0,80233893	0,4642927
TRIM2	0,88363058	0,6404183	0,84822237	0,4882152
TRIM44	0,80465864	0,51349034	0,91120475	0,52596814
TRPC4	0,67957291	0,35637272	0,87699381	0,44176358
TRPS1	0,73038632	0,4756899	0,77772223	0,40723051
TSPAN15	1,11004739	0,49842976	0,97849305	0,47227905
TSPAN5	0,73525817	0,39869023	0,77769154	0,48188225
UBE2E2	0,67170082	0,38109384	0,68161457	0,42701602
USP13	0,85357159	0,40998238	0,80765329	0,48602151
USP25	0,76249483	0,45105215	0,91347376	0,4945246
VPS54	0,87559937	0,45185769	0,92040069	0,49078541
WDR70	0,7964015	0,39910979	0,81841843	0,47533842
WIPI1	0,77114679	0,48755598	0,84413146	0,41416617
WNT5B	0,70152179	0,27186592	0,76816505	0,44140209
XPO7	0,79521046	0,48852822	0,90587118	0,46570844
ZCCHC7	0,75826922	0,42996864	0,87049394	0,48686012
ZFAT	0,70974412	0,43929381	0,82513634	0,49652356
ZFPM2	0,69672541	0,39470216	0,98540865	0,47586275
ZHX3	0,76314227	0,43801739	0,82805712	0,48558871
ZNF362	1,03794858	0,49126485	0,99161778	0,46645279
ZNF438	0,87819917	0,46766075	0,91009427	0,42084171
ZNF521	0,84194866	0,45008008	0,88235371	0,43816673
ZNF618	0,7326747	0,49728536	0,92875249	0,43467417
ZNF804A	0,75001962	0,42934684	1,03958433	0,45577304

Supplementary Table S2. Phenotype of the patient's cells according to the Laugel nomenclature[1]. The CS (I, II, III) classification was done according to the degree of severity of the disease.

Patient ID	Clinical subtype	Gene	Mutation 1	Mutation 2	Predicted protein for mutation 1	Predicted protein for mutation 2
AS852	CS III	CSA	c.478G>A	c.478G>A	p.Ala160Thr	p.Ala160Thr
AS466	CS I	CSA	c.173+1119G>C	c.173+1119G>C	p.0	p.0
AS655	CS I	CSA	c.582G>C	c.70dupA	p.Trp194Cys	p.Thr24fs
VL3	CS II	CSA	c.752delT	c.752delT	p.Leu251fs	p.Leu251fs
VL11	CS I	CSA	c.400-2A>G	c.400-2A>G	p.Thr134fs	p.Thr134fs
VL13	CS I	CSA	c.316C>T	c.316C>T	p.Gln106*	p.Gln106*
VL35	CS I	CSA	c.316C>T	c.316C>T	p.Gln106*	p.Gln106*
VL37	CS I	CSA	c.618-1G>A	c.356C>T	p.?	p.Ser119Leu
AS784	CS I	CSB	c.202_211del	c.2038A>G	p.Arg68fs	p.Asn680Asp
AS539	CS I	CSB	c.(?_-79)_14-?del	c.(?_-79)_14-?del	p.0	p.0
AS179	CS II	CSB	c.1248dupA	c.(?_-79)--14-?del	p.Val417fs	p.0
AS360	CS II	CSB	c.2060C>T	c.2867_2870delAAGT	p.Ser687Leu	p.Gln956fs
AS514	CS II	CSB	c.2287-2A>G	c.2287-2A>G	p.Val763_Gln794del	p.Val763_Gln794del
AS683	CS II	CSB	c.2170-1G>A	c.2058G>C	p.Val724_Gln762del	p.Trp686Cys
AS797	CS II	CSB	c.2960T>C	c.2254A>G	p.Leu987Pro	p.Met752_Gln762del
AS393	CS III	CSB	c.544-1G>A	c.1135G>T	p.Glu182fs	p.Glu379*
AS493	CS I	CSB	c.708G>A	c.1499delC	p.Trp236*	p.Pro500fs
AS177	CS II	CSB	c.2599_26A>G	c.3591_3592dupGA	p.Met867fs	p.Lys1198fs
AS548	CS I	CSB	c.(?_-79)--14-?del	c.(?_-79)--14-?del	p.0	p.0
AS385	CS II	CSB	c.2287-2A>G	c.2287-2A>G	p.Val763_Gln794del	p.Val763_Gln794del
AS823	CS III	CSB	c.3778+2T>G	c.2203C>T	p.Ser1240_Val1260delinsIle	p.Arg735*
AS543	CS III	CSB	c.1913A>G	c.2247delT	p.Arg637fs	p.Asp749fs
VL1	COFS	CSB	c.2047C>T	c.2047C>T	p.Arg683*	p.Arg683*
VL4	CS III	CSB	c.3778+2T>G	c.2203C>T	p.Ser1240_Val1260delinsIle	p.Arg735*
VL6	CS I	CSB	c.653-2A>G	c.653-2A>G	p.Glu218fs	p.Glu218fs
VL9	COFS	CSB	c.2612T>C	c.3513dupT	p.Leu871Pro	p.Lys1172*
VL12	CS II	CSB	c.1834C>T	deletion exon 1 and 2	p.Arg612*	p.0
VL34	COFS	CSB	c.1258C>T	c.3259C>T	p.Gln420*	p.Arg1087*
VL38	CS II	CSB	c.1971_1974dup	c.1971_1974dup	p.Thr659fs	p.Thr659fs

Supplementary Table S3. Summary of statistical support to **Supplementary Figure S1d.**

	CDK5RAP2		NRG1		NIPBL	
	CSA	WT	CSA	WT	CSA	WT
**Moyenne**	**0,456571**	**1,081204**	**0,526455**	**1,102464**	**0,527815**	**1,187895**
Variance	0,0340036	0,0348838	0,0208111	0,0168921	0,0240723	0,0058109
Observations	6	4	6	4	6	4
Variance pondérée	0,0343337		0,0193415		0,0172243	
Différence hypothétique des moyennes	0		0		0	
Degré de liberté	8		8		8	
Statistique t	-5,2224067		-6,4163745		-7,791692	
P(T<=t) unilatéral	0,0004001		0,0001028		2,639E-05	
Valeur critique de t (unilatéral)	1,859548		1,859548		1,859548	
**P(T<=t) bilatéral**	**0,0008**		**0,000206**		**5,28E-05**	
Valeur critique de t (bilatéral)	2,3060041		2,3060041		2,3060041	
	CSB	WT	CSB	WT	CSB	WT
**Moyenne**	**0,413369**	**1,081204**	**0,54849**	**1,102464**	**0,549385**	**1,187895**
Variance	0,010836	0,0348838	0,0182432	0,0168921	0,0150258	0,0058109
Observations	23	4	23	4	23	4
Variance pondérée	0,0137218		0,0180811		0,01392	
Différence hypothétique des moyennes	0		0		0	
Degré de liberté	25		25		25	
Statistique t	-10,523896		-7,6048278		-9,9898817	
P(T<=t) unilatéral	5,664E-11		2,921E-08		1,636E-10	
Valeur critique de t (unilatéral)	1,7081408		1,7081408		1,7081408	
**P(T<=t) bilatéral**	**1,13E-10**		**5,84E-08**		**3,27E-10**	
Valeur critique de t (bilatéral)	2,0595386		2,0595386		2,0595386	
	XPCS	WT	XPCS	WT	XPCS	WT
**Moyenne**	**0,284587**	**1,081204**	**0,199267**	**1,102464**	**0,35957**	**1,187895**
Variance	0,0508896	0,0348838	0,017963	0,0168921	0,0348065	0,0058109
Observations	4	4	4	4	4	4
Variance pondérée	0,0428867		0,0174276		0,0203087	
Différence hypothétique des moyennes	0		0		0	
Degré de liberté	6		6		6	
Statistique t	-5,4400543		-9,6756306		-8,2200558	
P(T<=t) unilatéral	0,0008011		3,494E-05		8,749E-05	
Valeur critique de t (unilatéral)	1,9431803		1,9431803		1,9431803	
**P(T<=t) bilatéral**	**0,001602**		**6,99E-05**		**0,000175**	
Valeur critique de t (bilatéral)	2,4469119		2,4469119		2,4469119	
	CSA	non CS	CSA	non CS	CSA	non CS
**Moyenne**	**0,456571**	**1,08424**	**0,526455**	**1,10267**	**0,527815**	**1,060056**
Variance	0,0340036	0,0267259	0,0208111	0,0699282	0,0240723	0,029387
Observations	6	15	6	15	6	15
Variance pondérée	0,0286411		0,0570027		0,0279884	
Différence hypothétique des moyennes	0		0		0	
Degré de liberté	19		19		19	
Statistique t	-7,6779887		-4,9963056		-6,5861554	
P(T<=t) unilatéral	1,534E-07		4,008E-05		1,323E-06	
Valeur critique de t (unilatéral)	1,7291328		1,7291328		1,7291328	
**P(T<=t) bilatéral**	**3,07E-07**		**8,02E-05**		**2,65E-06**	
Valeur critique de t (bilatéral)	2,0930241		2,0930241		2,0930241	
	CSB	non CS	CSB	non CS	CSB	non CS
**Moyenne**	**0,413369**	**1,08424**	**0,54849**	**1,10267**	**0,549385**	**1,060056**
Variance	0,010836	0,0267259	0,0182432	0,0699282	0,0150258	0,029387
Observations	23	15	23	15	23	15
Variance pondérée	0,0170154		0,0383429		0,0206107	
Différence hypothétique des moyennes	0		0		0	
Degré de liberté	36		36		36	
Statistique t	-15,496563		-8,5275909		-10,717993	
P(T<=t) unilatéral	8,344E-18		1,826E-10		4,718E-13	
Valeur critique de t (unilatéral)	1,6882977		1,6882977		1,6882977	
**P(T<=t) bilatéral**	**1,67E-17**		**3,65E-10**		**9,44E-13**	
Valeur critique de t (bilatéral)	2,028094		2,028094		2,028094	

Supplementary protocol. Molecular diagnostic test for CS patients using qRT-PCR

- Control gene for qRT-PCR normalization: *GAPDH* (NM_002046)
- ATF3 dependent genes to analyze: *CDK5RAP2* (NM_018249; NM_001011649), *NRG1* (NM_001160004; NM_001160008), *NIPBL* (NM_133433; NM_015384)
- compare gene expression profile of patient cell lines to controls

a) normal fibroblast non-transformed
b) CS-A fibroblast non-transformed and/or
c) CS-B fibroblast non-transformed

Conditions:

Standard fibroblast growth medium: DMEM/HAM-F10 (1:1) + 10% FCS + Gentamycin. To be adjusted if necessary.

Dish size: 10cm cell culture dishes with 10ml growth medium
Amount of cells: seed 2 x 10^6 cells
Confluency at time of irradiation: 80%
Irradiation: 10J/m^2 UV-C (254 nm)
4 time points: $\mathbf{T_{0h}}$ (no UV-C control), $\mathbf{T_{1h}}$, $\mathbf{T_{4h}}$, $\mathbf{T_{24h}}$ (hours after UV-C treatment)

Treatment of cells

- Aspirate all medium from cell dishes
- Wash once with pre-warmed 1x PBS
- Aspirate all PBS from cell dishes (very important, otherwise the UV radiation will not fully and equally penetrate to the cells → cant the plates so that all PBS can run to one side)
- Place cell dishes in the UV box and take off the lids (very important since lids are opaque, otherwise the UV radiation will not penetrate to the cells)
- Put fresh 10 ml of medium to the cell dishes and incubate for the appropriate time

RNA Isolation (QIAGEN RNeasy Mini Kit #74106)

- Work at room temperature
- Work under a hood
- Don´t spin in a cooled centrifuge
- Use e.g. RNase-Zap to decontaminate bench and pipettes
- Prepare DNase buffer mix: 70 µl RDD buffer + 10 µl DNase per sample, keep on ice (RNase-free DNase Set # 79254)

- Freeze down scratched cells in 1000 µl RLT buffer + 10 µl beta-Mercaptoethanol in a **2 ml** Eppendorf tube
- Defreeze lysate at room temperature (best in your palm)
- Vortex briefly
- Heating block for 15 to 20 min at 37°C
- Vortex and spin down briefly

- Mix lysate at equal volume with 70% ethanol
- Load 650 µl lysate/EtOH mix to a spin column and spin at **10.000 rpm for 1 min**
- Discard flow-through and repeat step until all lysate/EtOH mix is loaded onto the column
- Change 2 ml collection tube
- load 350 µl buffer RW1 onto the column and spin at **10.000 rpm for 1 min**
- Discard flow-through
- Load 80 µl of DNase buffer mix directly onto the column and let stand for 30 min at RT
- load 350 µl buffer RW1 onto the column and spin at **10.000 rpm for 1 min**
- Discard flow-through
- load 500 µl buffer RPE onto the column and spin at **10.000 rpm for 1 min**
- Discard flow-through
- load 500 µl buffer RPE onto the column and spin at **10.000 rpm for 2 min**
- Discard flow-through
- Spin column dry at **14.000 rpm for 4 min**
- Transfer column into a clean 1,5 ml tube and load 30 - 50 µl of RNase-free water directly onto the column and let stand for 10 min at RT
- spin at **10.000 rpm for 1 min**
- discard column
- transfer eluate quickly to ice
- measure RNA concentration with Nanodrop system
- measure 1,5 µl of the RNA eluate
- Flash freeze your samples in liquid N_2 (-196°C) and store at -80°C

cDNA synthesis (Invitrogen SuperScript II Reverse Transcriptase #18064-014)

- ➢ 500 ng of total RNA per cDNA
- ➢ Take RNA in triplicate
- ➢ From each RNA sample produce three cDNA samples
- ➢ use Oligo $(dT)_{15}$ primer at 500 µg/ml ➔ 100 µM stock concentration ➔ 100 pmoles/µl ➔ 495 µg/ml ssDNA ➔ 1 µl of undiluted stock solution
- ➢ use dNTPmix at 10 mM each ➔ 10 µl of each dNTP (100 mM stock solution) + 60 µl RNase-free water

1) mix on ice:

Oligo $(dT)_{15}$	1 µl
total RNA (500 ng)	x µl
dNTPmix	1 µl
water	fill to 12 µl

2) heat samples for 5 min at 65°C, transfer samples back to ice
3) spin down briefly
4) add to each sample:

First-strand buffer 5x 4 µl	
DTT 0,1 M	2 µl

5) heat samples for 2 min at 42°C, transfer samples back to ice
6) add to each sample:
SS II RT 1 µl

7) incubate samples for 50 min at 42°C
8) inactivate reaction by heating for 15 min at 70°C
9) spin down briefly
10) add to each sample:
Ribonuclease H 1 µl (Promega #M4281)

11) incubate samples for 20 min at 37°C
12) spin down briefly

Given the cDNA synthesis performs at 100% efficiency the concentration should be at 25 ng/µl.

qRT-PCR (QIAGEN QuantiTect SYBR Green PCR Master Mix #204145)

- prepare a standard dilution curve:
- take 10 µl from each sample from triplicate #1 and mix them in one tube
- dilution steps: undiluted (1), 1:10, 1:100, 1:1.000, 1:10.000
- take 10 µl from undiluted cDNA mix and mix it with 90 µl water (1:10)
- take 10 µl from 1:10 cDNA mix and mix it with 90 µl water (1:100)
- take 10 µl from 1:100 cDNA mix and mix it with 90 µl water (1:1000)
- take 10 µl from 1:1000 cDNA mix and mix it with 90 µl water (1:10.000)
- for qRT-PCR make 1:50 dilutions (5 µl cDNA sample with 245 µl H_2O) of all cDNA samples
- use white 96-well qPCR plates
- analyze triplicates on same plate
- fragment to amplify should be approx. 100 bp long

- set up a 20 µl reaction volume:

qPCR Master-Mix	10 µl
Primer forward (10 µM)	1µl
Primer rev (10 µM)	1µl
cDNA	3µl
H_2O	5µl

- seal 96-well qPCR plates with a transparent seal foil
- spin down plate
- qPCR conditions on Roche LC 480

15min at 95˚C Pre-heating
15sec at 95˚C
15sec at 60˚C 55 cycles
25sec at 72˚C

Name: ___________________________

Date of RNA extraction: ___________________________

Timepoint	conc. (ng/µl)	$E_{260/280}$	$E_{260/230}$	vol. for 500 ng RNA	vol. water	dilution for cDNA
1 T_0						
1 T_1						
1 T_4						
1 T_{24}						
2 T_0						
2 T_1						
2 T_4						
2 T_{24}						
3 T_0						
3 T_1						
3 T_4						
3 T_{24}						
4 T_0						
4 T_1						
4 T_4						
4 T_{24}						
5 T_0						
5 T_1						
5 T_4						
5 T_{24}						
6 T_0						
6 T_1						
6 T_4						
6 T_{24}						

Name: ___________________________

Date of evaluation: ___________________________
